# Climatic Changes and Vegetation Responses During Holocene Characteristic Period in the Northeastern Qinghai–Tibet Plateau

**DOI:** 10.3390/life15040572

**Published:** 2025-04-01

**Authors:** Huayong Zhang, Hao Chen, Yihe Zhang, Zhongyu Wang, Zhao Liu

**Affiliations:** 1 Research Center for Engineering Ecology and Nonlinear Science, North China Electric Power University, Beijing 102206, China; chanho1@foxmail.com (H.C.); zhy_wang@ncepu.edu.cn (Z.W.); 2 Theoretical Ecology and Engineering Ecology Research Group, School of Life Sciences, Shandong University, Qingdao 250100, China; 15540959555@163.com; 3 School of Engineering, RMIT University, P.O. Box 71, Melbourne, VIC 3083, Australia; henin.zhang@rmit.edu.au

**Keywords:** Holocene characteristic period, climate change, vegetation response, weighted averaging–partial least squares method, northeastern Qinghai–Tibet Plateau

## Abstract

The Qinghai–Tibet Plateau represents a highly sensitive region to global climate change. Understanding Holocene climate variations and vegetation responses in this area holds significant value for predicting future climate patterns, vegetation distribution changes, and biodiversity loss. Here, we aim to reconstruct Holocene climate conditions in the northeastern Qinghai–Tibet Plateau using modern and Holocene pollen records through weighted averaging–partial least squares (WA-PLS) analysis, and to examine the spatial–temporal relationship between vegetation dynamics and climate change during different characteristic periods. The results indicate that: (1) During the Holocene, the climate generally tended toward warmth and humidity with increased extremity. Based on temperature variations, it can be divided into ten characteristic periods. (2) The Holocene saw an increase in maximum temperature, average temperature, and precipitation, while minimum temperature decreased. (3) Forest decreased, undergoing three stages: expansion, contraction, and continuous contraction leading to stabilization. Grassland increased, following the stages of full expansion, localized expansion, and contraction with stabilization. (4) Under climatic influence, forest areas slightly expanded, while grassland areas slightly contracted. Meanwhile, land salinization intensified. We aim to enhance the understanding of climate change and vegetation evolution, providing a theoretical basis for addressing future climate change and biodiversity loss.

## 1. Introduction

Global warming has profoundly impacted vegetation dynamics and biodiversity on a global scale [1,2,3]. Reconstructing paleoclimate and paleovegetation is essential for deciphering historical climate change patterns and elucidating the complex response relationships between vegetation and climatic conditions [4,5,6,7,8,9,10,11]. Such reconstructions are particularly valuable because analyzing ecosystem cycles enables the prediction of future natural trends and environmental shifts [12]. Furthermore, investigating the causes of extreme climate events in Earth’s history can help identify analogous conditions that may trigger ecological collapse in modern ecosystems [13,14,15]. Additionally, validating climate models through paleoclimate reconstructions provides a robust foundation for refining predictive tools, thereby enhancing the accuracy of future climate change simulations [16,17,18,19].

Pollen analysis has become a cornerstone in paleoenvironmental studies due to three key advantages: (1) ubiquitous preservation—pollen grains are widely distributed and well-preserved in sediments across diverse ecosystems; (2) climate sensitivity—different pollen types have distinct climatic preferences; (3) quantifiable relationships—modern pollen-climate databases enable precise reconstructions [20,21,22,23,24]. Over time, pollen-based paleoclimate research has advanced significantly, it has transitioned from early qualitative studies that linked increases in specific pollen types to broad climatic changes [25,26], to the use of pollen ratios for more refined climatic indicators [27,28], and now to sophisticated quantitative reconstructions employing transfer functions, such as weighted averaging–partial least squares (WA-PLS)—a method conceptually similar to how facial recognition algorithms predict age by analyzing key features, but in this case, extracting past climate signals from pollen assemblages [29].

The Qinghai–Tibet Plateau serves as an ideal natural laboratory for studying global climate change [30]. The uplift of the plateau has profoundly influenced the formation of monsoon systems and the distribution of precipitation. Existing research on the plateau has primarily focused on the quantitative reconstruction of climatic indicators during the Holocene using pollen data [31]. However, there remains a need for more precise delineation of characteristic periods [32] and a deeper understanding of the relationship between vegetation and climate [33]. By identifying characteristic periods of the Holocene based on temperature variations and cold/warm events, and employing isopleth mapping to study vegetation–climate interactions in the northeastern Qinghai–Tibet Plateau—a region experiencing pronounced climatic fluctuations—we can uncover the long-term impacts of climate change on ecosystems and elucidate the adaptive mechanisms of plants to climatic shifts.

In this study, we utilize modern climate records, along with modern and ancient pollen records from the northeastern Qinghai–Tibet Plateau, to reconstruct Holocene climate indicators using the weighted averaging–partial least squares (WA-PLS) method. Based on these reconstructions, we classify the Holocene into distinct stages and analyze spatial and temporal patterns of climate and vegetation changes. Finally, we investigate the response of vegetation to climatic variations. The specific research objectives are as follows: (1) to classify characteristic periods of the Holocene based on average temperature variations and cold/warm events; (2) to reconstruct the spatiotemporal pattern of climate change using contour maps; and (3) to analyze the spatiotemporal pattern of vegetation change through contour maps and explore the vegetation–climate response relationship using pollen ratio analysis. The overarching aim of this study is to enhance our understanding of climate change and vegetation evolution during the Holocene, thereby providing a theoretical foundation for addressing contemporary challenges related to climate change and biodiversity loss.

## 2. Materials and Methods

### 2.1. Data Collection and Processing

**Figure 1:**
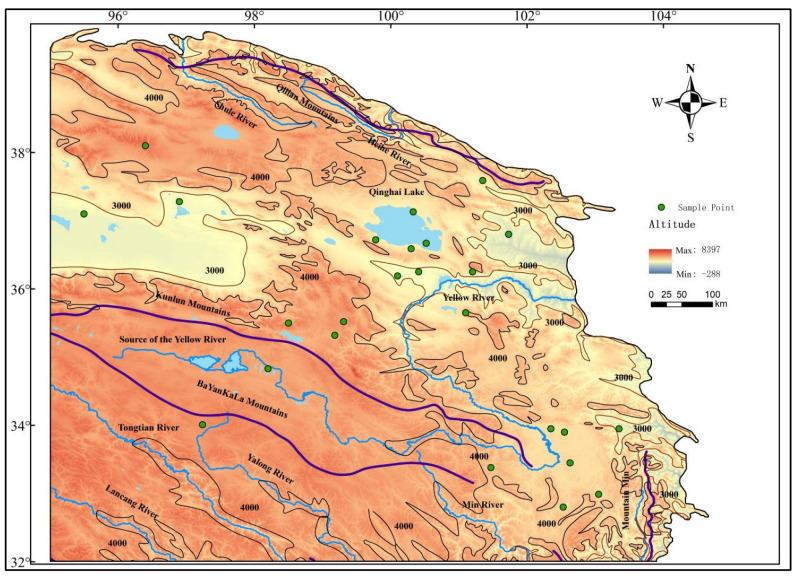
Contour map and research point distribution in the northeast of Qinghai–Tibet Plateau.

The study area is located on the Qinghai–Tibet Plateau, spanning 95°~104°47′ E and 32°~39°47′ N (Figure 1). The base map of the study area was derived from the China Landscape Map (1:30,000,000) http://bzdt.ch.mnr.gov.cn/index.html (accessed on 1 December 2024). Data on mountains, rivers, and elevation were obtained from the National Tibetan Plateau Data Center https://data.tpdc.ac.cn (accessed on 15 November 2024). Vegetation data were sourced from the Vegetation Map of the People’s Republic of China (1:1,000,000) www.plantplus.cn/dsite/zhibei/b12.html (accessed on 16 November 2024). Both ancient and modern pollen data were acquired from the National Tibetan Plateau Data Center [34].

The dataset comprises 254 ancient pollen sampling points, of which 28 located within the study area were selected for analysis. Similarly, from a total of 9165 modern pollen sampling points, 239 located within the study area were chosen as representative modern data. Pollen types accounting for less than 1.5% of the total were excluded from the analysis. The remaining pollen types in the dataset include: Abies, Betula, Picea, Pinus, Artemisia, Asteraceae, Chenopodiaceae, Cyperaceae, Ephedra, and Poaceae. Collectively, these ten pollen types represent approximately 80–90% of the total pollen composition.

Modern climate data were sourced from the WorldClim2.1 database, including four climate indicators: minimum temperature, maximum temperature, average temperature, and precipitation. Due to the sparse distribution of data points, this paper chose 20 km × 20 km resolution data for climate reconstruction during the Holocene.

### 2.2. Paleoclimate Reconstruction Methods

**Figure A1:**
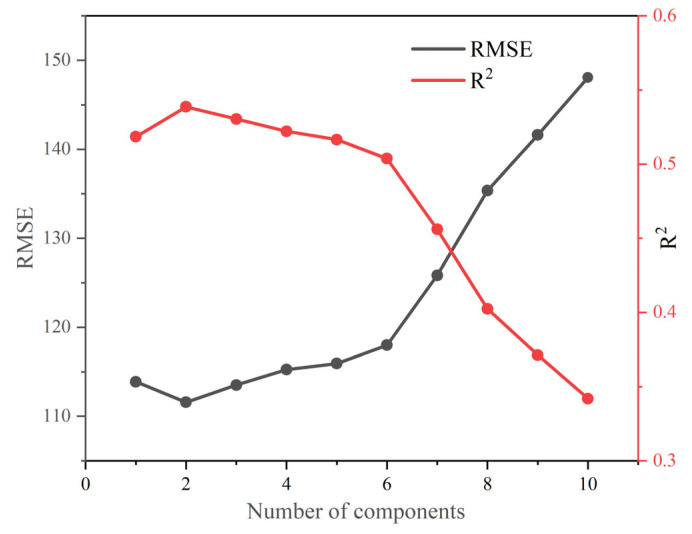
Scree plot of the WA-PLS modern calibration model (239 surface pollen samples across the Qinghai–Tibet Plateau).

We applied weighted averaging–partial least squares (WA-PLS) [35], a widely-used statistical method for translating pollen data into climate variables. The analysis was conducted in R 4.3.2 using the “rioja” package, following four key steps: (1) Data Standardization: All variables (modern pollen, climate data, and Holocene pollen) were scaled to have a mean of 0 and standard deviation of 1. This ensures equal weighting among variables with different units. (2) Model Construction: We built 10 candidate models with increasing complexity (1–10 principal components). Adding components improves fit but risks overfitting. (3) Model Selection (Figure A1): The optimal model was chosen by balancing: prediction accuracy (highest R^2^) and generalizability (lowest RMSE via 10-fold cross-validation). (4) Holocene Climate Reconstruction: Holocene pollen data were input to the optimal model to estimate past climate.

Based on the fluctuation trends of mean temperature, the Holocene was divided into several characteristic periods. Spatial climate patterns were analyzed using ArcGIS software version 10.7, with Kriging interpolation applied to climate data from each characteristic period. The study focused on the target region, generating isoline maps for minimum temperature, maximum temperature, mean temperature, and precipitation.

While WA-PLS relies on the assumption that modern pollen–climate relationships apply to the past (a limitation when no modern analogs exist), it outperforms the traditional Modern Analog Technique (MAT) by utilizing weighted averaging and partial least squares regression to extract continuous climate signals from entire datasets, making it the most accurate and widely-used quantitative paleoclimate reconstruction method. The latest WA-PLS developments feature Monte Carlo-based uncertainty quantification [36], Huber loss functions for outlier robustness, and asymmetric weighting to improve extreme-climate pollen response modeling [37]. Additionally, since our dataset and WA-PLS reconstructions only include climatic indices without physical, chemical, or biological proxies, comparisons with independent climate records may introduce certain errors.

### 2.3. Calculation of Vegetation Response

Given that Generalized Additive Models (GAMs) may produce unstable results with our limited sample size (*n* < 100) and are sensitive to potential outliers in paleoclimate records, we selected Pearson correlation analysis to assess pollen sensitivity to climate. Using Origin software, yielding correlation coefficients (r) and corresponding *p*-values, a *p*-value < 0.05 was considered indicative of a statistically significant relationship between pollen and climate variables. The vegetation response to climate was further evaluated by comparing the proportions of pollen types exhibiting varying degrees of significant correlations with climate parameters (Table A1).

In this study, several pollen ratios were utilized to infer ecological and environmental changes. The Artemisia/Chenopodiaceae (A/C [26]) ratio was used to assess shifts in forest versus grassland coverage. The Picea/Betula (Pi/Be) ratio was employed to determine the development of forest types, distinguishing between coniferous and deciduous dominance. The Chenopodiaceae/Ephedra (Ch/Ep) ratio was applied to infer the degree of land salinization or alkalization. The Artemisia/Poaceae (Ar/Po) ratio was used to evaluate changes in the dominance of Artemisia versus Poaceae within grassland ecosystems.

The spatial patterns of ancient pollen and pollen ratios were calculated using ArcGIS software. The contour mapping method was consistent with that used for climate contours. Within the study area, contour maps were generated separately for forest pollen, grassland pollen, and each pollen ratio to analyze their spatial distributions.

## 3. Results

### 3.1. Holocene Characteristic Period and Climate Change

**Figure 2:**
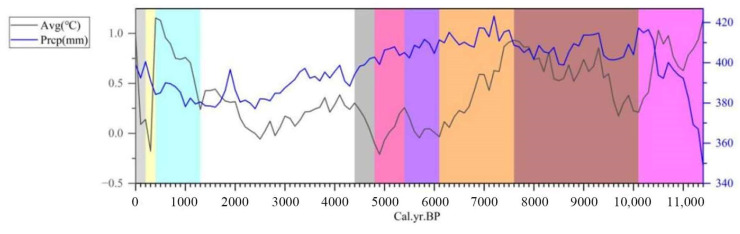
Average temperature and precipitation variations during the Holocene.

During the Holocene, the climate exhibited a general trend toward warmer and more humid conditions, accompanied by increasing climatic variability and extremes. The reconstructed temperature ranges were as follows: minimum temperatures varied between −6.84 °C and −5.33 °C, maximum temperatures between 6.42 °C and 8.31 °C, and mean temperatures between −0.21 °C and 1.15 °C (Figure 2). Precipitation levels fluctuated from 348 mm to 423 mm during the same period (Figure 2). Based on the observed temperature trends, the Holocene can be divided into ten distinct periods, categorized as follows: Warming periods: 10.1~7.6 ka BP, 6.1~5.4 ka BP, 4.8~4.4 ka BP, 1.3~0.4 ka BP, and 0.2 ka BP to the present. Cooling periods: 11.4~10.1 ka BP, 7.6~6.1 ka BP, 5.4~4.8 ka BP, and 0.4~0.2 ka BP. Stable temperature periods: 4.4~1.3 ka BP. This division highlights the dynamic nature of Holocene climate, characterized by alternating phases of warming, cooling, and relative stability.

**Figure 3:**
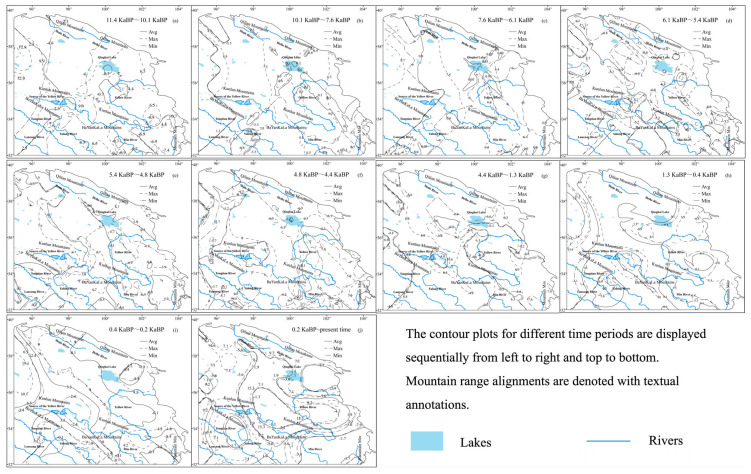
Temperature contour maps during the Holocene.

During the Holocene, temperatures exhibited significant fluctuations, with an overall warming trend. The mean temperature increased by 0.27 °C, while the maximum temperature rose by 4.42 °C and the minimum temperature dropped by 5.88 °C. Low-temperature zones were predominantly concentrated near Mountain Min and Qinghai Lake. However, by the late Holocene, a new low-temperature region emerged near the Tongtian River, with all three areas displaying comparable minimum temperature values. The lowest recorded temperature of −8.4 °C occurred during 0.4~0.2 ka BP, while the highest minimum temperature of −6 °C was observed during 7.6~6.1 ka BP. High-temperature zones were primarily located in the eastern Qinghai Lake region and the northern Kunlun Mountains, later shifting to the central Kunlun Mountains. The lowest high-temperature value of 8.7 °C was recorded during 7.6~6.1 ka BP, while the highest value of 19.1 °C occurred during 0.4~0.2 ka BP. The range of mean temperatures was narrowest at 0.87 °C during 7.6~6.1 ka BP and widest at 8.3 °C during 0.4~0.2 ka BP (Figure 3).

**Figure 4:**
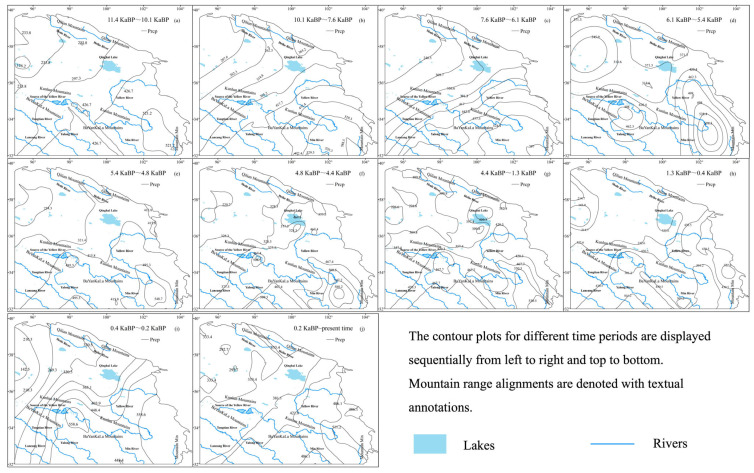
Precipitation contour maps during the Holocene.

During the Holocene, precipitation exhibited a relatively stable trend, initially increasing before gradually decreasing, with an overall rise of 56.7 mm. High-precipitation zones were predominantly concentrated near Mountain Min, while arid regions were primarily located in the northern Kunlun Mountains. Precipitation reached its peak of 588.5 mm during 10.1~7.6 ka BP and declined to a minimum of 142.9 mm during 0.4~0.2 ka BP (Figure 4). The changes in precipitation can be divided into three distinct phases: a rapid increase from 11.4 to 7.2 ka BP, a slow decrease from 7.2 to 5 ka BP, and a fluctuating decline from 5 ka BP to the present. Spatially, precipitation distribution showed no significant expansion trend, remaining relatively stable over time. However, the disparity in precipitation between the northwest and southeast regions diminished during this period.

### 3.2. Vegetation Response

**Figure 5:**
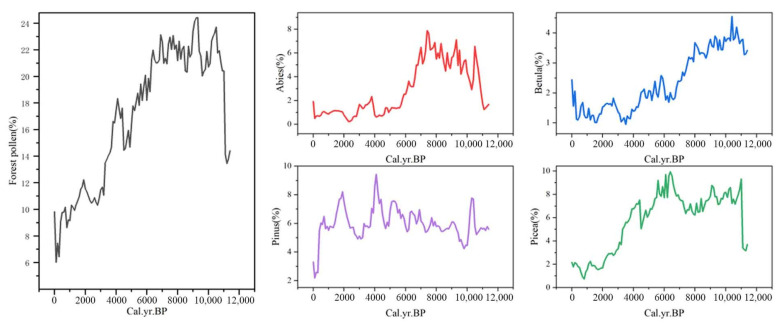
Forest pollen and pollen type variations during the Holocene.

During the Holocene, forest coverage remained relatively low. Forest pollen exhibited an initial increase followed by a gradual decline, with an overall reduction of 4.58% (Figure 5). Among the dominant tree taxa, Abies pollen percentages ranged from 0.23% to 7.88% and showed a primarily positive correlation with maximum temperature. In contrast, Betula pollen percentages ranged from 0.95% to 4.55% and were predominantly negatively correlated with minimum temperature. Picea pollen percentages varied between 0.73% and 9.94%, displaying a strong positive correlation with precipitation. Similarly, Pinus pollen percentages ranged from 2.18% to 9.42% and were also mainly positively correlated with precipitation (Table A1).

**Figure 6:**
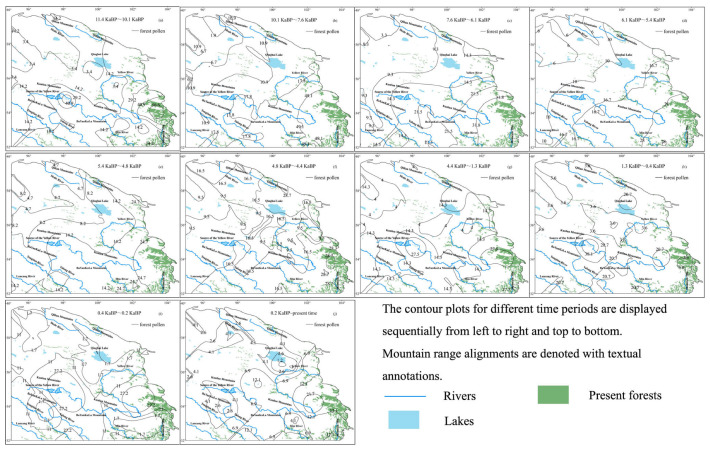
Forest pollen contour maps during the Holocene.

During the Holocene, forests were primarily concentrated near the Mountain Min and the central Kunlun Mountains. However, by the end of this period, forested areas near the Kunlun Mountains significantly diminished, resulting in a distribution pattern that closely resembles modern forest coverage. Forest pollen proportions peaked at 56.6% during 11.4~10.1 ka BP but declined to a low of 20.7% during 1.3~0.4 ka BP (Figure 6). Forest expansion occurred from 11.4 to 7.6 ka BP, followed by a gradual retreat from 7.6 to 4.4 ka BP. From 4.4 ka BP to the present, forests continued to shrink, ultimately shaping the contemporary distribution pattern observed today.

**Figure 7:**
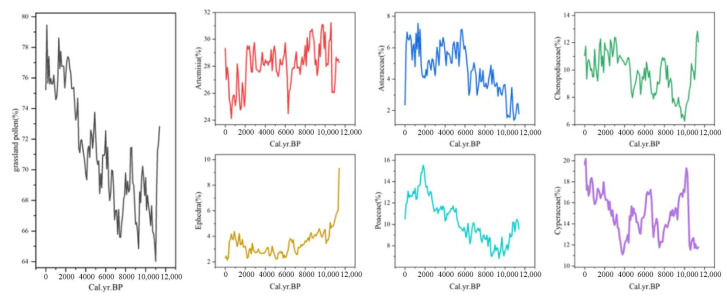
Grassland pollen and pollen type variations during the Holocene.

During the Holocene, grassland vegetation was dominant, with grassland pollen showing an overall increasing trend of 2.39% and consistently ranging between 60% and 80% (Figure 7). Among the key grassland taxa, Asteraceae pollen percentages ranged from 1.39% to 7.55% and were primarily negatively correlated with minimum temperature. Poaceae pollen percentages ranged from 6.82% to 15.54% and were mainly negatively correlated with maximum temperature. Artemisia pollen percentages ranged from 24.12% to 31.23% and were predominantly negatively correlated with precipitation. Chenopodiaceae pollen percentages ranged from 6.26% to 12.8% and were mainly positively correlated with minimum temperature. Cyperaceae pollen percentages ranged from 11.09% to 20.20% and were primarily negatively correlated with maximum temperature. Finally, Ephedra pollen percentages ranged from 2.15% to 9.31% and were mainly positively correlated with maximum temperature (Table A1).

**Figure 8:**
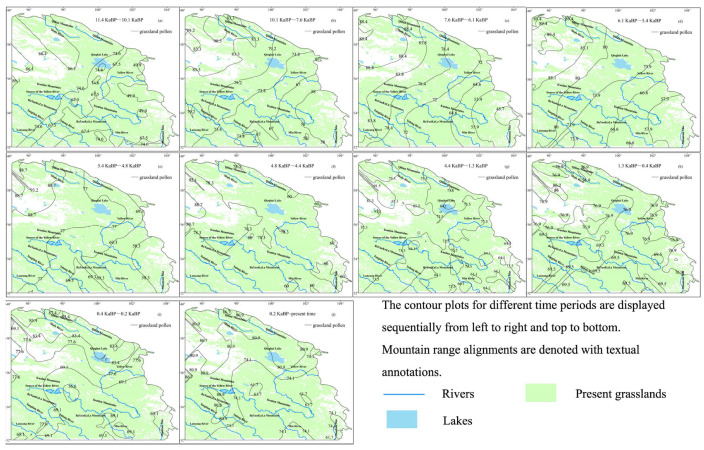
Grassland pollen contour maps during the Holocene.

Grassland vegetation was primarily concentrated near the Qilian Mountains during the Holocene. By the end of this period, its extent near the Qilian Mountains had diminished, while its proportion and scale increased near Mountain Min. This shift resulted in a higher proportion of grassland pollen near Qinghai Lake, the Yellow River, the Kunlun Mountains, and the Tongtian River. Grassland pollen proportions peaked at 93.2% during 4.8~4.4 ka BP and reached a minimum of 83.4% during 0.4~0.2 ka BP (Figure 8). Grassland expansion occurred in two phases: a full expansion from 11.4 to 7.6 ka BP, followed by localized expansion from 7.6 to 4.4 ka BP. From 4.4 ka BP to the present, grasslands underwent retraction, ultimately forming the contemporary distribution pattern observed today.

**Figure 9:**
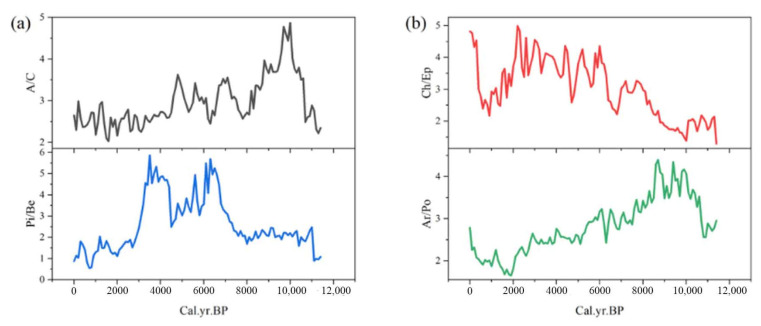
Pollen ratio variations during the Holocene: (**a**) A/C and Pi/Be, (**b**) Ch/Ep and Ar/Po.

**Figure 10:**
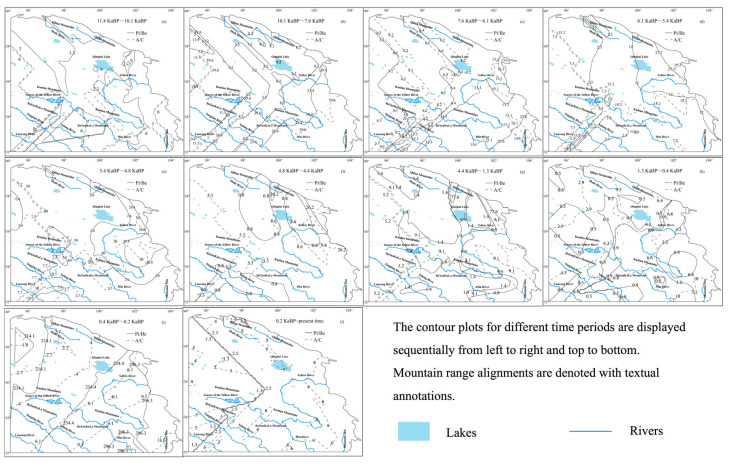
Pi/Be and A/C contour maps during the Holocene.

The Pi/Be ratio experienced an initial stable phase, followed by a mid-period increase and a late-period decline, with an overall decrease of 0.2. Meanwhile, the A/C ratio showed an early increase, a mid-period decrease, and a late-period stabilization, with an overall increase of 0.3 (Figure 9). These trends suggest an overall development of vegetation toward forested ecosystems, with a shift within forests from coniferous to deciduous dominance. The Pi/Be ratio decreased from the southeast to the northwest, indicating a gradual transition from coniferous to deciduous forests along this gradient. The ratio reached its maximum value of 296.3 during 0.4~0.2 ka BP, corresponding to the period of the fastest coniferous forest expansion. Conversely, the minimum value of 2.5 occurred during 0.2 ka BP to the present, marking the period of the fastest deciduous forest expansion. Higher A/C values were predominantly observed south of the Yellow River and west of the Kunlun Mountains, reflecting a shift from grassland to forest in these regions. The A/C ratio peaked at 39.6 during 10.1~7.6 ka BP, coinciding with the period of the fastest forest expansion, while its minimum value of 8.6 occurred during 4.8~4.4 ka BP, corresponding to the period of the fastest grassland expansion (Figure 10).

**Figure 11:**
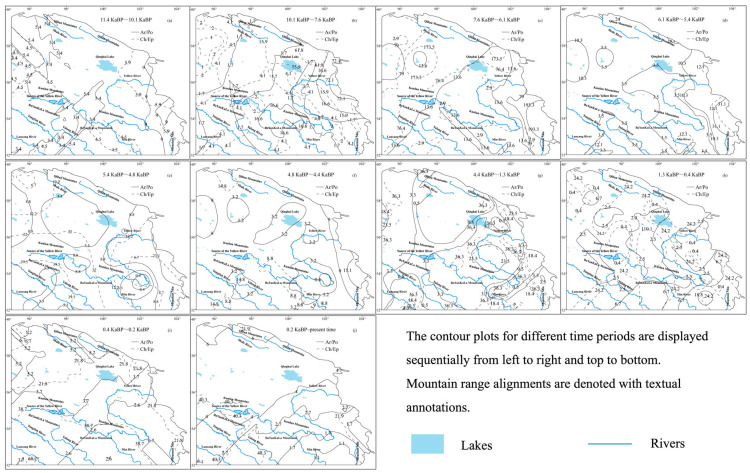
Ch/Ep and Ar/Po contour maps during the Holocene.

The Ar/Po (Artemisia/Poaceae) ratio initially increased and then decreased, with an overall reduction of 0.17, indicating a shift in grassland composition toward Poaceae dominance. In contrast, the Ch/Ep (Chenopodiaceae/Ephedra) ratio showed a consistent increase, rising by 3.52 (Figure 9), suggesting an intensification of land salinization over time. Higher Ar/Po values are primarily observed in the northwest and southeast regions, reflecting the dominance of Artemisia plants in these areas. The Ar/Po ratio reached its maximum value of 72.1 during 10.1~7.6 ka BP, corresponding to the period of fastest Artemisia expansion, while its minimum value of 5.2 occurred during 0.4~0.2 ka BP, marking the period of the fastest Poaceae expansion. Higher Ch/Ep values are mainly concentrated in the northern Kunlun Mountains and near Mountain Min, indicating the most severe land salinization and alkalization in these regions. The Ch/Ep ratio peaked at 173.5 during 7.6~6.1 ka BP, when salinization was the most severe, and reached its minimum value of 12.1 during 6.1~5.4 ka BP, when salinization was the least pronounced (Figure 11).

## 4. Discussion

Existing studies [38,39,40,41,42,43,44,45] suggest that the Holocene can be broadly divided into 11 characteristic periods based on distinct cold and warm events, 11.4 ka BP~10 ka BP cooling, 10.0 ka BP~8.9 ka BP warming, 7.9 ka BP~7.4 ka BP cooling, 7.2 ka BP~6.0 ka BP warming, 5.9 ka BP~5.5 ka BP cooling, 5.5 ka BP~4.0 ka BP warming, 4.2 ka BP~2.8 ka BP cooling, 1.7 ka BP~1.3 ka BP cooling, 0.81 ka BP~0.48 ka BP warming, 0.4 ka BP~0.2 ka BP cooling, 0.2 ka BP~present warming. In this study, as the climatic fluctuations in the later stage of the Holocene were relatively mild, we identified 10 characteristic periods based on climatic features. While the temperature trends align closely with previous research, the starting times and durations of some periods exhibit differences. Notably, three periods are consistent with established findings: (1) the cooling period from 11.4 to 10.1 ka BP, attributed to the lingering effects of the Younger Dryas event [46]; (2) the Little Ice Age from 0.4 to 0.2 ka BP; and (3) the warming period from 0.2 ka BP to the present, driven by the Industrial Revolution.

The prolonged warming period from 10.1 to 7.6 ka BP was driven by the intensification of the Asian monsoon [47], influenced by the thermal effects of the Qinghai–Tibet Plateau. The subsequent 8.2 ka BP cold event [48] indirectly impacted the plateau through the melting of Northern Hemisphere ice sheets, resulting in a delayed cooling period from 7.6 to 6.1 ka BP. This delay caused a later onset and shorter duration of the 6.1–5.4 ka BP period in our results, which differs from Shi’s findings [39]. Furthermore, later Ice Rafted Debris (IRD) layer events [49] indirectly affected the Qinghai–Tibet Plateau, leading to shifts in the timing of characteristic periods. The period from 4.4 to 1.3 ka BP did not exhibit significant cooling compared to existing studies, but IRD events at 4.1 ka BP, 2.7 ka BP, and 1.4 ka BP triggered abrupt cooling episodes, aligning the temperature trends more closely with current research. The warming period from 1.3 to 0.4 ka BP began earlier than previously documented, likely due to a temperature rebound following the end of the 1.4 ka BP IRD event. Spatially, this study identified a trend of southward shifts in both low-temperature and high-temperature centers, suggesting that natural climate variability may have contributed to global warming and increased climate instability.

Precipitation during the Holocene was significantly influenced by monsoon dynamics. According to studies by Chen [50] and Lv [51], Holocene precipitation changes can be divided into three distinct stages. From 11.4 to 7 ka BP, precipitation increased steadily under the influence of the monsoon, reaching its maximum levels. From 7 to 5 ka BP, the intensity of the East Asian Summer Monsoon (EASM) peaked, sustaining high regional precipitation, although a slight decline was observed. From 5 ka BP to the present, the weakening of both the EASM and the Indian Summer Monsoon (ISM) led to a general decrease in precipitation, accompanied by significant fluctuations. The results of this study align broadly with these findings, though after 2.4 ka BP, while precipitation exhibited an overall declining trend, it began to show notable fluctuations. This variability may be attributed to the influence of various cold and warm events during the Holocene. Spatially, the precipitation center remained consistent, concentrated near Mountain Min, but the precipitation gradient between the southeast and northwest regions has diminished. This easing of spatial differences may reflect more evenly distributed precipitation patterns resulting from global warming.

During the Holocene, forests underwent significant contraction. According to studies by Wende Z [52], Herzschuh [53], and Sun [54], the Qinghai–Tibet Plateau experienced forest expansion from 11.7 to 5.3 ka BP, with coniferous forests dominating during this period. From 6.0 to 2.0 ka BP, forest areas drastically decreased. Since 4.6 ka BP, coniferous forests have notably retreated, indicating that forest development during the Holocene followed three distinct stages: early expansion, mid-period contraction, and late retreat and stabilization. The findings of this study align closely with those of previous researchers. When combined with modern forest distribution patterns, it is evident that contemporary forests gradually formed during the late Holocene. Spatially, forests initially had only two distribution centers, located near Mountain Min and the Kunlun Mountains. By the mid-Holocene, a new distribution center emerged near Qinghai Lake. However, by the late Holocene, only Mountain Min remained as a significant distribution center, reflecting a clear trend of southward forest migration.

During the Holocene, grasslands experienced a slight overall expansion, but land salinization worsened significantly. According to studies by Tao Wang [55], Guangliang [56], Feiya Lv [57], and Huang [58], grassland coverage on the Qinghai–Tibet Plateau reached its maximum of 47.2% at 8.7 ka BP, with grasslands and meadows expanding further from 11.4 to 6.0 ka BP. From 8.0 to 4.5 ka BP, grasslands continued to expand, and vegetation distribution became more diverse, with Artemisia becoming the dominant species. Since 4.7 ka BP, grasslands have rapidly degraded due to the combined effects of climate change and human activities. This suggests that grassland development during the Holocene followed three distinct stages: early expansion, sustained expansion in the mid-Holocene, and late degradation and stabilization. The results of this study align broadly with those of other researchers, though the degradation of grasslands in the third stage was less pronounced. This is because, during the late Holocene, both grasslands and forests were degrading, and the decline in forests reduced their competitive pressure on grasslands, leading to localized degradation and expansion. Additionally, a distinct distribution pattern emerged in the late Holocene, with grasslands primarily concentrated around the Qilian Mountains, Kunlun Mountains, and Qinghai Lake, closely resembling modern grassland distribution. Initially, the distribution center was near the Qilian Mountains, but it expanded to the northern Kunlun Mountains during the mid-Holocene. By the late Holocene, the distribution center in the northern Kunlun Mountains disappeared, while grassland areas near Mountain Min increased. This reflects a trend where northern grasslands were suppressed, while southern grasslands were promoted.

## 5. Conclusions

The Qinghai–Tibet Plateau is a critical region for studying global climate change. Investigating climate change and vegetation response during the Holocene on the plateau provides valuable insights for addressing future climate change and biodiversity loss. This study utilized Holocene pollen data from 28 sites and modern pollen and climate data from 930 sites, to reconstruct Holocene climate data for the 28 sites using the weighted averaging–partial least squares (WA-PLS) method. Based on the average temperature fluctuations, the Holocene was divided into ten characteristic periods. By mapping climate and vegetation contours for each period, the spatial and temporal patterns of vegetation and climate changes were analyzed. Pearson correlation analysis was used to assess the sensitivity of pollen to climate, and four pollen ratios (A/C, Pi/Be, Ar/Po, Ch/Ep) were applied to evaluate vegetation responses to climate. The key findings are as follows: (1) Holocene Climate Periods: The Holocene can be divided into ten periods based on climate characteristics: Cooling and increased humidity occurred during 11.4~10.1 ka BP, 7.6~6.1 ka BP, and 0.4~0.2 ka BP; warming and reduced humidity occurred during 10.1~7.6 ka BP, 6.1~5.4 ka BP, 4.8~4.4 ka BP, and 0.2 ka BP to the present; cooling and reduced humidity occurred during 5.4~4.8 ka BP; little change was observed during 4.4~1.3 ka BP; warming and increased humidity occurred during 1.3~0.4 ka BP. (2) Holocene Climate Trends: The overall climate trend during the Holocene was characterized by warming and increased humidity. The average temperature rose by 0.27 °C, the maximum temperature increased by 4.42 °C, and the minimum temperature decreased by 5.88 °C. Precipitation increased by 56.7 mm, and the maximum temperature difference widened by 13.7 °C. Low-temperature areas near Qinghai Lake and Mountain Min did not expand significantly, but a new low-temperature area emerged near the Tongtian River by the end of the Holocene. High-temperature areas, primarily concentrated around the Kunlun Mountains, shifted from the northern to the central regions. Precipitation trends exhibited three phases: a rapid increase from 11.4 to 7.2 ka BP, a slow decline from 7.2 to 5 ka BP, and a fluctuating decrease from 5 ka BP to the present. While there was no significant expansion of precipitation areas, regional differences in precipitation diminished. (3) Precipitation was the primary driver of forest dynamics. The proportion of forest pollen decreased by 4.58%, indicating a reduction in forest vegetation. Forests were mainly located near Mountain Min and underwent three phases: expansion from 11.4 to 7.6 ka BP, retraction from 7.6 to 4.4 ka BP, and continued retraction and stabilization from 4.4 ka BP to the present. Maximum temperature was the main driver of grassland dynamics. The proportion of grassland pollen increased by 2.39%, reflecting an expansion of grassland vegetation. Grasslands were primarily distributed near the Qilian Mountains, Kunlun Mountains, and Qinghai Lake, experiencing three phases: full expansion from 11.4 to 7.6 ka BP, localized expansion from 7.6 to 4.4 ka BP, and retraction and stabilization from 4.4 ka BP to the present. (4) A/C increased by 0.3, indicating a shift toward forest dominance; Pi/Be decreased by 0.2, reflecting a transition from coniferous to deciduous forests; Ch/Ep increased by 3.52, suggesting intensified soil salinization; Ar/Po decreased by 0.17, indicating a shift toward Poaceae dominance in grasslands. These results demonstrate that, under climatic influence, forests slightly expanded (dominated by deciduous forests), while grasslands slightly contracted (dominated by Poaceae). Soil salinization intensified during the Holocene. This study provides a scientific basis for predicting future climate changes and addressing biodiversity loss.

## Data Availability

All links to input data are reported in the manuscript and all output. Data are available upon request to the authors.

## Appendix B

**Table A1:** Results of Pearson correlation analysis (r-value: Correlation Coefficient, *p*-value: Statistical Significance).

Pollen	Tmax	Tmin	Avg	Prcp
Abies	r = 0.657	-	r = 0.412	r = 0.456
	*p* < 0.0001	-	*p* < 0.0001	*p* < 0.0001
Betula	-	r = −0.190	-	-
	-	*p* = 0.042	-	-
Picea	-	r = −0.444	-	r = 0.609
	-	*p* < 0.0001	-	*p* < 0.0001
Pinus	r = 0.764	r = 0.448	r = 0.665	r = 0.722
	*p* < 0.0001	*p* < 0.0001	*p* < 0.0001	*p* < 0.0001
Artemisia	r = −0.183	-	-	r = −0.460
	*p* = 0.0497	-	-	*p* < 0.0001
Asteraceae	-	r = −0.438	r = −0.341	-
	-	*p* < 0.0001	*p* = 0.000191	-
Chenopodiaceae	-	r = 0.447	-	r = −0.415
	-	*p* < 0.0001	-	*p* < 0.0001
Cyperaceae	r = −0.237	-	r = −0.188	-
	*p* = 0.0108	-	*p* = 0.0447	-
Ephedra	r = 0.263	-	-	-
	*p* = 0.00445	-	-	-
Poaceae	r = −0.207	-	-	-
	*p* = 0.0265	-	-	-
